# Identification of key metabolic enzymes involved in the activation of obeldesivir and remdesivir to the active triphosphate metabolite

**DOI:** 10.1128/aac.00055-26

**Published:** 2026-06-22

**Authors:** Yili Xu, Albert Liclican, Xiaofeng Zhao, Chen Chen, Joseph T. Ortega, Hsiu-Chun Chuang, Cameron Soulette, Subhra Chaudhuri, Jingyu Zhang, Ting Wang, Darius Babusis, Bruno Marchand, Kai-hui Sun, Ricardo Ramirez, Bin Ma, Arabinda Nayak, Liya Wang, Saurabh Menon, Jean-Philippe Belzile, Yurong Lai, Li Li, Richard L. Mackman, John P. Bilello, Tomas Cihlar, Joy Y. Feng, Latesh Lad

**Affiliations:** 1 Gilead Sciences, Inc. 2158 , Foster City, California, USA; 2 Department of Animal Biosciences, Swedish University of Agricultural Sciences 8095, Uppsala, Sweden; Chinese Academy of Medical Sciences & Peking Union Medical College, Beijing, China

**Keywords:** obeldesivir, ODV, phosphotransferases, activation pathway, GS-441524

## Abstract

Obeldesivir (GS-5245, ODV), an orally administered 5′-isobutyryl ester prodrug of GS-441524, has demonstrated anti-SARS-CoV-2 activity in preclinical and clinical settings. ODV is hydrolyzed in plasma to provide high systemic exposures of GS-441524, enabling the efficient intracellular formation of the bioactive 5′-triphosphate GS-443902, an ATP analog inhibiting the SARS-CoV-2 RNA-dependent RNA polymerases. Remdesivir (RDV), the first US FDA-approved antiviral treatment for COVID-19, is also activated to GS-443902. In this paper, we systematically profiled the human enzymes potentially involved in ODV and RDV activation using multiple approaches: (i) biochemical studies on the catalytic efficiency (*k*_cat_/*K*_m_); (ii) analysis of activation in single-gene knockout cells; and (iii) the antiviral activity assessment in single-gene knockout cells. Our results demonstrated that ODV hydrolysis to GS-441524 is catalyzed by carboxylesterase 1 and 2 with high efficiency. For the enzymes involved in the consecutive formation of 5′-mono-, di-, and tri-phosphates (MP, DP, and TP), adenosine kinase (ADK) likely plays a minor role in forming GS-441524-MP, suggesting the involvement of other phosphotransferases. This was further supported by cell-based single-gene ADK-knockout studies. In contrast, both biochemical and cell-based assay data strongly support adenylate kinase 2 as the key enzyme for the phosphorylation of GS-441524-MP, while nucleoside diphosphate kinase NM23-H2, phosphoglycerate kinase, and pyruvate kinase likely all contribute to the formation of GS-441524-TP. This work leads to a deeper understanding of ODV and RDV metabolism, and suggests further studies are needed to identify the enzyme responsible for the activation of GS-441524 to its 5′-monophosphate.

## INTRODUCTION

The COVID-19 pandemic has been the largest global pandemic since the 1918–1920 influenza outbreak and led to more than 778 million cases, and an estimated 7.1 million deaths based on WHO reports (reference https://covid19.who.int (accessed 5th January 2026). After many years, COVID-19 cases and mortality rates have finally declined significantly, driven by successful vaccination, effective antiviral therapies, and widespread population immunity. As a result, the United States federal COVID-19 Public Health Emergency ended in May 2023. However, SARS-CoV-2 variants will continue to present public health challenges worldwide for years or even decades (1). In addition, long COVID or chronic COVID syndrome, the residual symptoms lasting 4 weeks or longer after acute COVID-19 illness, has as high as 43% global prevalence and demands better prevention and treatment options (2, 3).

Despite the promising advances in treatment, limitations exist regarding these first-generation treatment options. Remdesivir (RDV), a nucleotide prodrug that targets the SARS-CoV-2 RNA-dependent RNA polymerase (RdRp), requires IV administration by healthcare professionals. Nirmatrelvir is an inhibitor of the SARS-CoV-2 3CL protease co-administered with the P450 3A4 inhibitor ritonavir as Paxlovid, which is contraindicated in some patients due to drug-drug interactions (DDIs), and symptom rebounds have been reported (https://www.covid19treatmentguidelines.nih.gov/therapies/antivirals-including-antibody-products/ritonavir-boosted-nirmatrelvir--paxlovid-/paxlovid-drug-drug-interactions/ ). Molnupiravir (Lagevrio), a nucleoside analog that targets SARS-CoV-2 RdRp by inducing lethal mutations during viral RNA synthesis, has raised concerns about potential off-target toxicity (4).

Recently, we reported the discovery of obeldesivir (GS-5245, ODV), a 5′-isobutyryl ester prodrug of GS-441524 with therapeutic antiviral activity in SARS-CoV-2-infected African green monkey (AGM) (5), in SARS-CoV-2, SARS-CoV, MERS-CoV-infected mice models (6), SARS-CoV-2-infected ferrets (7), and recently in a Phase III clinical trial (8). The active intracellular metabolite of ODV is the 5′-triphosphate (TP) of GS-441524, GS-443902, the same active triphosphate formed by metabolism of RDV, and an ATP analog that inhibits SARS-CoV-2 RdRp replication and transcription of viral RNA (9, 10)

Like RDV, ODV belongs to a class of nucleoside/tide analogs that play important roles in anti-viral and anti-cancer therapies (11–15). Like other nucleosides, GS-441524 cellular uptake is not a rate-limiting step for its phosphorylation/activation, and this has been well studied in the field. Cellular uptake can influence GS-441524 activation in some physiological contexts, but it is generally not considered the primary rate-limiting step for intracellular triphosphate formation (16). Once inside the cell, nucleosides need to be transformed into their active 5′-triphosphate form by cellular or viral kinases to exert their pharmacological activity (17–24). Through three consecutive steps, nucleoside analogs are phosphorylated to the respective 5′-monophosphate (MP), 5′-diphosphate (DP), and finally 5′-triphosphate (TP). The formation of the MP metabolite is often the slowest (rate-limiting) step, and the discovery of monophosphate prodrugs has fundamentally improved the nucleoside/tide antiviral therapy by circumventing this step to TP formation (13). Previously, we reported three hydrolases, including cathepsin A (CatA), carboxylesterase 1 (CES1), and histidine triad nucleotide-binding protein 1 (HINT1) that play key roles in the initial metabolic activation of RDV in human lung cells to GS-441524-MP (25). However, little is known about the enzymes involved in the three consecutive but biochemically distinct steps constituting the series of the following conversions: GS-441524 → GS-441524-MP, GS-441524-MP → GS-441524-DP, and GS-441524-DP → GS-441524-TP (GS-443902). Additionally, there is limited information about the hydrolase(s) that cleaves ODV systemically and pre-systemically to produce high plasma exposures of GS-441524, which ultimately produce therapeutic levels of intracellular GS-443902.

**Fig 1:**
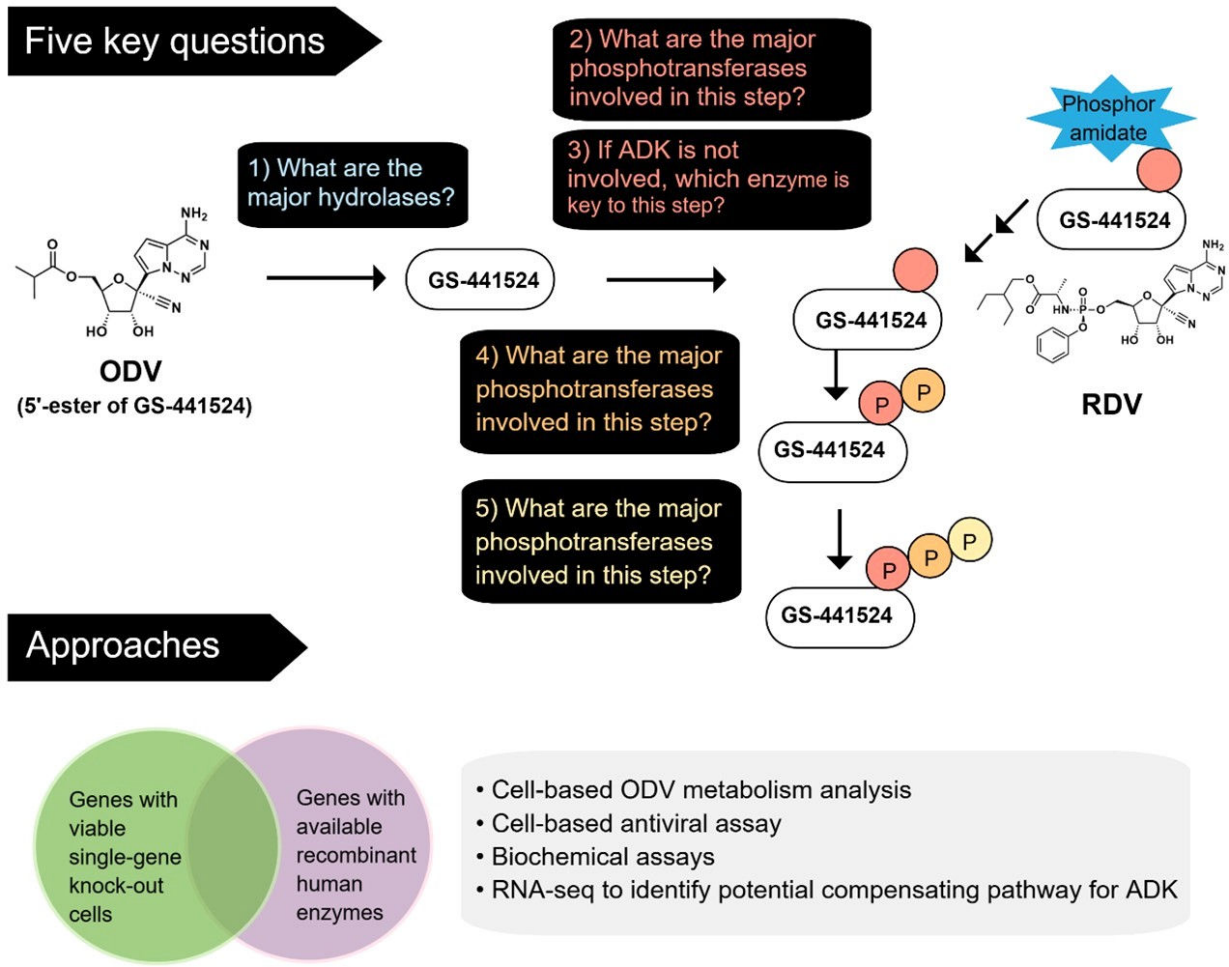
Five key questions raised in this study and approaches to address them.

**Fig 2:**
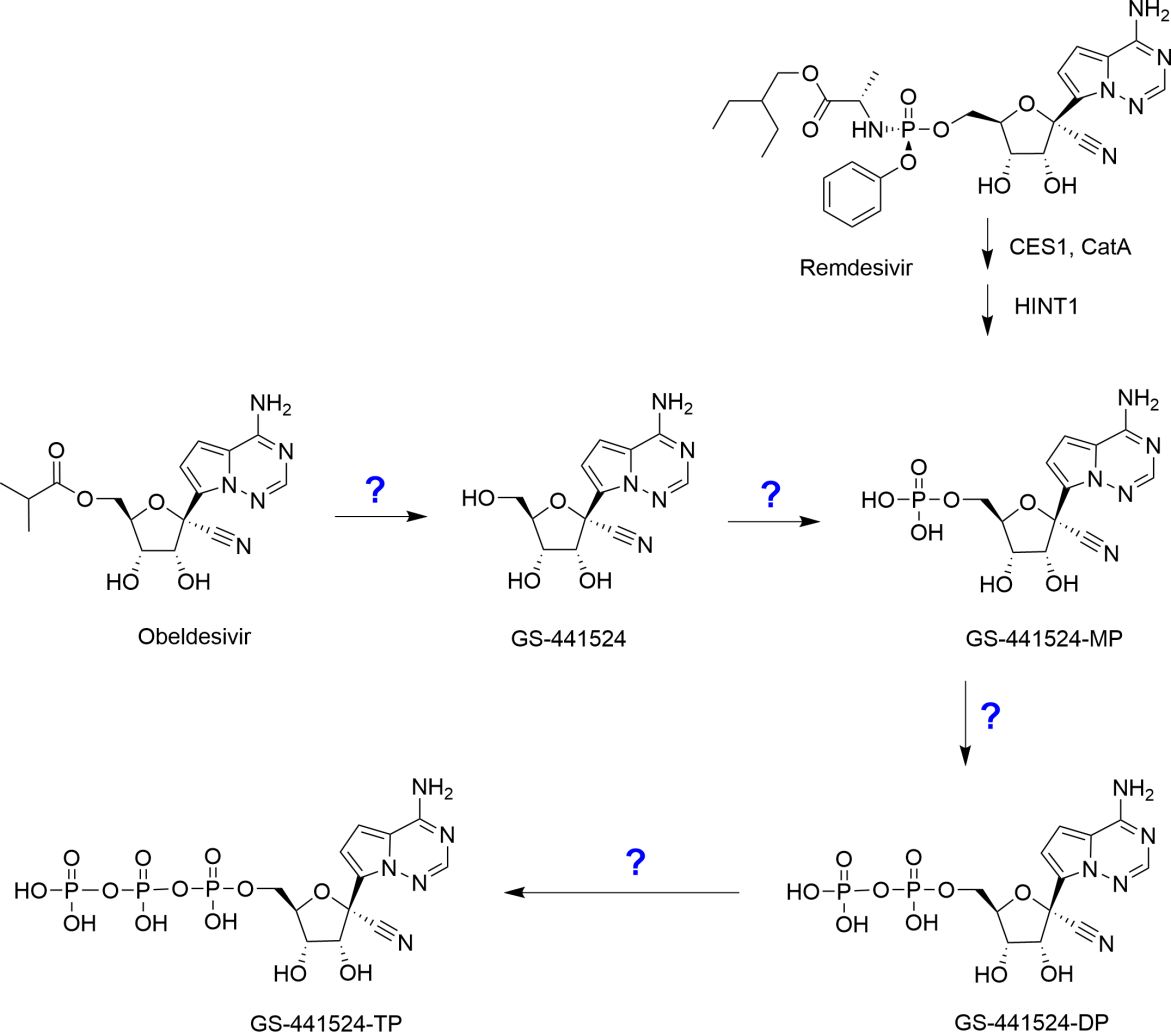
Activation pathway of obeldesivir and remdesivir with unknown metabolic enzymes highlighted in question marks.

In this report, we aim to address key metabolic questions as illustrated in Fig. 1, that inform on the activation of ODV to its active TP metabolite, using multiple approaches: (i) biochemical studies with recombinant human enzymes to measure catalytic efficiencies (*k*_cat_/*K*_m_) of the analog phosphorylation and compare to that of their natural substrates; (ii) evaluation of ODV or GS-441524 activation in single-gene knockout cells; and (iii) assessment of ODV or GS-441524 for their antiviral activities in single-gene knockout cells. We selected a broad panel of enzymes based on their documented capacity to metabolize nucleoside/nucleotide analogs (17, 20, 24). Since the activation of RDV from GS-441524-MP to -TP overlaps with that of the ODV activation pathway, this study also adds to our prior work (25) and completes profiling of the entire activation pathway of RDV. As outlined in Fig. 2, we aim to identify the key enzymes involved in the four biochemical transformations leading to the formation of the active metabolite GS-441524-TP: ODV to GS-441524, GS-441524 to GS-441524-MP, GS-441524-MP to GS-441524-DP, and finally, GS-441524-DP to GS-441524-TP.

## RESULTS

### Biochemical evaluation of potential ester hydrolases

We previously reported that ODV hydrolysis to GS-441524 is catalyzed by carboxylesterases CES1 and CES2 with a half-life (*t*_1/2_) less than 5 minutes (5). However, no detailed biochemical characterization was performed. In this study, we measured the hydrolysis rates using a previously published method (25). As summarized in Table 1, ODV is an efficient substrate for human CES1A1, CES1(BC012418), and CES2 enzymes with hydrolysis rates of 0.17, 0.21, and 1.34 nmol min^−1^ mg^−1^, respectively. However, ODV is not a substrate of cathepsin A (CatA), as shown by the low hydrolysis rate of <7.8 × 10^−4^ nmol min^−1^ mg^−1^. Our data further supported the notion that ODV is cleaved pre-systemically based on prior *in vitro* and *in vivo* pharmacokinetic studies (7, 16).

**TABLE 1:** Evaluation of potential hydrolases for the conversion of ODV to GS-441524^*c*^

Enzymes	ODV^*a*^	Controls	
	Rate of hydrolysis (nmol min^−1^ mg^−1^) [Substrate *T*_1/2_]		Rate of hydrolysis (nmol min^−1^ mg^−1^) [Substrate *T*_1/2_]
CatA	<7.8 × 10^−4^ [>592 min]	TAF	0.017 [27.9 min]
CES1A1^*b*^	0.17 [2.7 min]	Oseltamivir	0.025 [18.5 min]
CES1(BC012418)^*b*^	0.21 [2.2 min]	Oseltamivir	0.012 [37.6 min]
CES2	1.34 [0.35 min]	Procaine	3.8 x 10^−3^ [122.4 min]

^*a*^ All data were averaged from two independent experiments with technical duplicates.

^*b*^ CES1A1 and CES1(BC012418) are recombinant human CES1 proteins obtained from Discovery Life Sciences (DLS). The sequence of cDNA was sourced from NCBI GeneBank accession number BC110338 for CES1A1 (AB119997, https://doi.org/10.2133/dmpk.23.73) and BC012418 for CES1(BC012418). BC012418 encodes a CES1 variant with a coding sequence identical to CES1A2 but with a Q362 deletion.

^*c*^ The concentration of all substrates was tested at 1 µM.

### Biochemical evaluation of potential phosphotransferases

**Fig 3:**
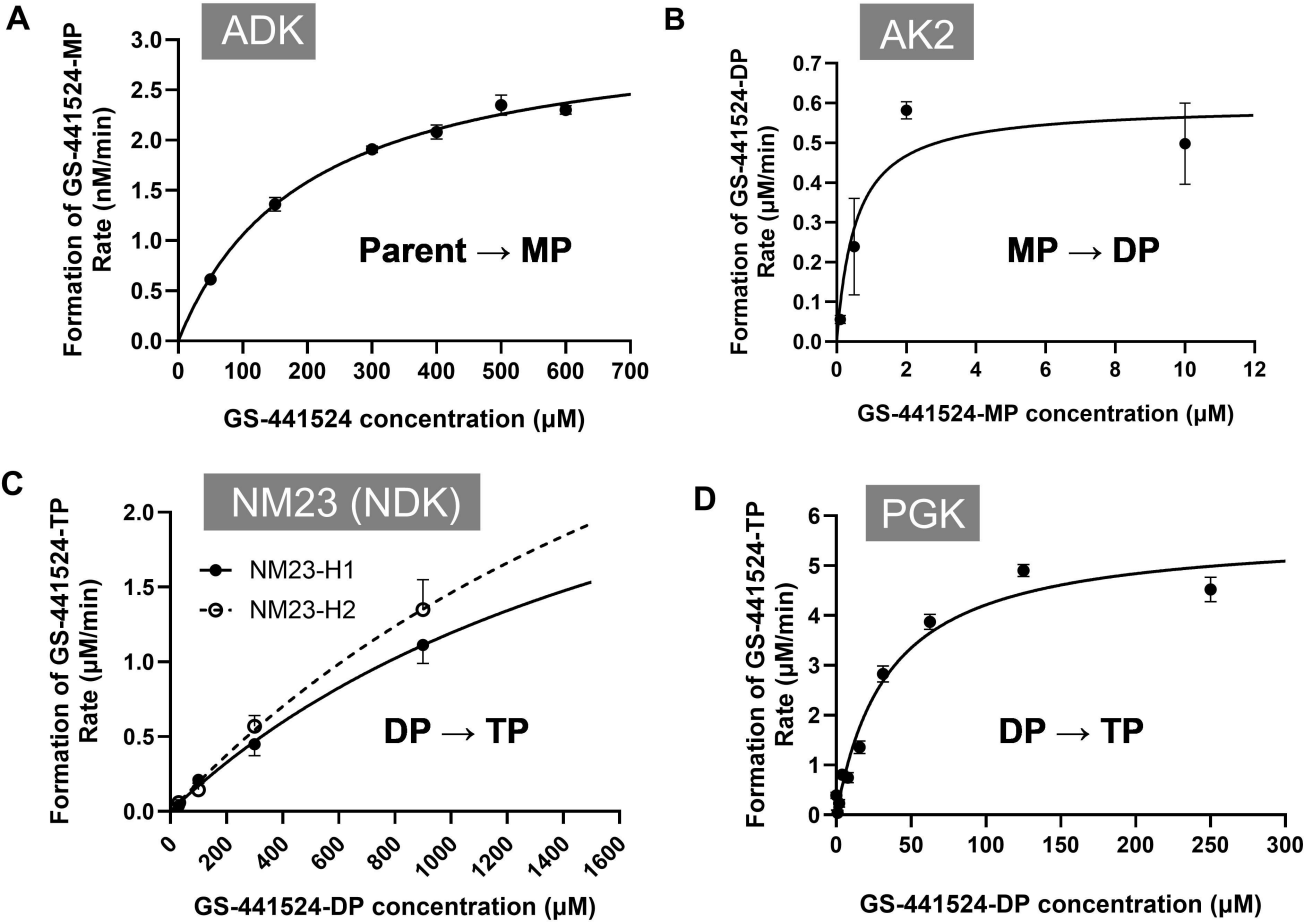
Representative *K*_m_ titration curves for the phosphorylation of GS-441524 (**A**), GS-441524-MP (**B**), and GS-441524-DP (**C and D**) to illustrate the significant differences in the catalytic rate (product formed nM [for ADK] or µM [for other enzymes] per min) and *K*_m_. The enzymes illustrated here are human ADK, AK2, and nucleotide diphosphate kinases (NDKs) NM23-H1 and NM23-H2, and PGK. (**A**) Michaelis-Menten curves of GS-441524 phosphorylation by human ADK. Note units of product formation are in nM, 1,000 times below those typically observed with the natural substrate adenosine; (**B**) Michaelis-Menten curves of GS-441524-MP phosphorylation by human AK2; (**C**) Michaelis-Menten curves of GS-441524-DP phosphorylation by human NDKs NM23-H1 (shown in closed circles) and NM23-H2 (shown in open circles); (**D**) Michaelis-Menten curves of GS-441524-DP phosphorylation by human PGK.

We selected a broad panel of recombinant human phosphotransferases known for their involvement in the purine/pyrimidine salvage pathway or the activation of nucleoside/tide analogs. We measured the steady-state kinetic parameters *K*_cat_ and *K*_m_ for each enzyme to evaluate their potential roles in transforming GS-441524, GS-441524-MP, and GS-441524-DP into the MP, DP, and TP metabolites, respectively. A few representative *K*_m_ titrations are shown in Fig. 3. All data are summarized in Table 2.

**TABLE 2:** Phosphorylation of GS-441524, MP, and DP by recombinant human phosphotransferases^*f*^

Enzymes and substrates for each step of the phosphorylation	*K*_m_^*a*^ (µM)	*k*_cat_ (min^−1^)	*k*_cat_/*K*_m_ (µM^−1^ min^−1^)	*K*_m_ (µM)	*k*_cat_ or [maximal *k*_obs_ in the range of substrate conc. tested] (min^−1^)	*k*_cat_/*K*_m_ (µM^−1^ min^−1^)	Relative catalytic efficiency^*b*^
**Nucleoside → MP**	Natural substrate^*c*^			GS-441524			
Adenosine kinase (ADK)	0.72	14.2	19.8	193 ± 5	0.0033 ± 0.0003	1.71 × 10^−5^	1.16 × 10^6^
Deoxycytidine kinase (dCK)	0.218	0.040	0.19	NA^*d*^	< 0.0024 [0–100 µM]	NA	[>17]
Inosine-guanosine kinase (IGK), guanosine as substrate	7.5	0.39	0.052	NA	<0.0064 [0–100 µM]	NA	[>62]
Deoxyguanosine kinase (dGK)	0.83	0.24	0.292	BLQ^*e*^	BLQ [0–400 µM]	NA	NA
Thymidine kinase 1 (TK1)	1.4	13.3	9.5	NA	<0.0028 [0–10 µM]	NA	[>4,670]
Thymidine kinase 2 (TK2)	8.1	6.9	0.85	NA	<0.024 [0–100 µM]	NA	[>290]
Uridine cytidine kinase 1 (UCK1), uridine as substrate	581	94.6	0.16	NA	<0.014 [0–100 µM]	NA	[>6,525]
Uridine cytidine kinase 2 (UCK2), uridine as substrate	78.6	2,219	28	NA	< 0.012 [0–100 µM]	NA	[>192,034]
High *K*_m_ 5′-nucleotidase (5′-NT), adenosine as substrate	>5,000	NA	NA	NA	BLQ [0–2,000 µM]	NA	NA
**MP → DP**	AMP			GS-441524-MP			
Adenylate kinase 1 (AK1)	756	1,497	1.98	BLQ	BLQ [0–1,000 µM]	NA	NA
Adenylate kinase 2 (AK2)	197	701	3.56	840	250	0.30	12
Guanylate kinase (GUK)	16	833	52	NA	2.8 [0–250 µM]	NA	[>300]
Thymidylate kinase (TMPK)	32.9	0.26	0.008	NA	0.004 [0–200 µM]	NA	[>66]
Cytidylate kinase (CK)	2,000	227	0.11	NA	0.12 [0–4750 µM]	NA	[>1,960]
**DP → TP**	ADP			GS-441524-DP			
NM23H1 (NDK A)	53	807	15	1,995	7,146	3.6	4.2
NM23H2 (NDK B)	43	1,371	32	2,605	10,530	4.0	8.0
Phosphoglycerate kinase (PGK)	15.4	3,655	237	35	227.3 [0–500 µM]	6.49	36
Creatine kinase MM (CKMM)	257	136	0.53	NA	<0.0062 [0–1,000 µM]	NA	[>21,930]
Creatine kinase MB (CKMB)	140	315	2.26	NA	<0.00062 [0–1,000 µM]	NA	[>509,225]
Pyruvate kinase M2 (PKM2)	300	26,970	90	433.5	978 [0–1,250 µM]	2.26	40
Pyruvate kinase LR (PKLR)	201	3,177	15.8	218.6	26.1 [0–1,250 µM]	0.12	132

^*a*^ All values are averages from at least two measurements.

^*b*^ Relative catalytic efficiency is calculated from (*k*_cat_/*K*_m_)^Natural substrate^/(*k*_cat_/*K*_m_)^ Analog^.

^*c*^ Natural substrates are used according to the enzyme’s name, unless it is ambiguous.

^*d*^ NA = not applicable, primarily due to the minimal level of product formed, often less than the level of detection.

^*e*^ BLQ = below the level of quantitation.

^*f*^ The steady-state kinetic parameters are reported as the Michaelis constant (*K*_m_), the catalytic rate constant *k*_cat_, and the catalytic efficiency (*k*_cat_/*K*_m_) for both the natural substrates and the analogs of interest, including GS-441524, GS-441524-MP, and GS-441524-DP. The relative catalytic efficiency is calculated from the ratio of the catalytic efficiency values, with a higher number indicating that the enzyme is more difficult to use the analog as a substrate. Please note that the calculations presented here do not account for other factors, such as variations in protein expression levels for each enzyme across different cells or organs.

We evaluated nine human phosphotransferases for their ability to phosphorylate GS-441524 to GS-441524-MP, including adenosine kinase (ADK), deoxycytidine kinase (dCK), inosine-guanosine kinases (IGK), deoxyguanosine kinase (dGK), thymidine kinase 1 (TK1), thymidine kinase 2 (TK2), uridine cytidine kinase 1 (UCK1), uridine cytidine kinase 2 (UCK2), and high *K*_m_ 5′-nucleotidase (5′-NT). Among these, only ADK exhibited measurable activity under the assay conditions. However, ADK-mediated phosphorylation of GS-441524 was approximately 1 × 1.16e6-fold less efficient than that of adenosine. This dramatic reduction resulted from a combined effect of a 4,300-fold decrease in catalytic rate constant *k*_cat_ - 14.2 min^−1^ for adenosine versus 0.0033 min^−1^ for GS-441524, and a 268-fold decrease in the binding affinity (or higher *K*_m_)—0.72 µM for adenosine versus 193 µM for GS-441524.

Five recombinant human phosphotransferases were evaluated for their ability to transform GS-441524-MP to GS-441524-DP, including adenylate kinase 1 (AK1), adenylate kinase 2 (AK2), guanylate kinase (GUK), thymidylate kinase (TMPK), and cytidylate kinase (CK). While GS-441524-MP was found to be a very poor substrate for AK1, which resulted in product formation under detection. AK2 was able to phosphorylate GS-441524-MP very efficiently, with only approximately 12-fold less activity than the natural substrate AMP. This resulted from a 2.8-fold slower rate constant (250 min^−1^ for GS-441524-MP versus 701 min^−1^ for AMP) and a 4.3-fold weaker *K*_m_ (840 µM for GS-441524-MP versus 197 µM for AMP).

For the biotransformation of GS-441524-DP to TP, we evaluated seven recombinant human phosphotransferases, including NM23H1 or nucleoside diphosphate kinase A (NDK A), NM23H2 or nucleoside diphosphate kinase B (NDK B), phosphoglycerate kinase (PGK), creatine kinase MM (CKMM), creatine kinase MB (CKMB), pyruvate kinase M2 (PKM2), and pyruvate kinase LR (PKLR). Five of these enzymes showed high catalytic efficiency (*k*_cat_/*K*_m_) toward GS-441524-DP relative to their natural substrate, in decreasing order: NDK A > NDK B > PGK ≥ PKM2 > PKLR. This is consistent with previously published work and demonstrates the promiscuous substrate specificity of these phosphotransferases (24).

### Generation of single-gene KO cell lines, isolation of single clones of ADK- and AK2-KO cells, and their effects on ODV and GS-441524 activation and antiviral effects

**Fig 4:**
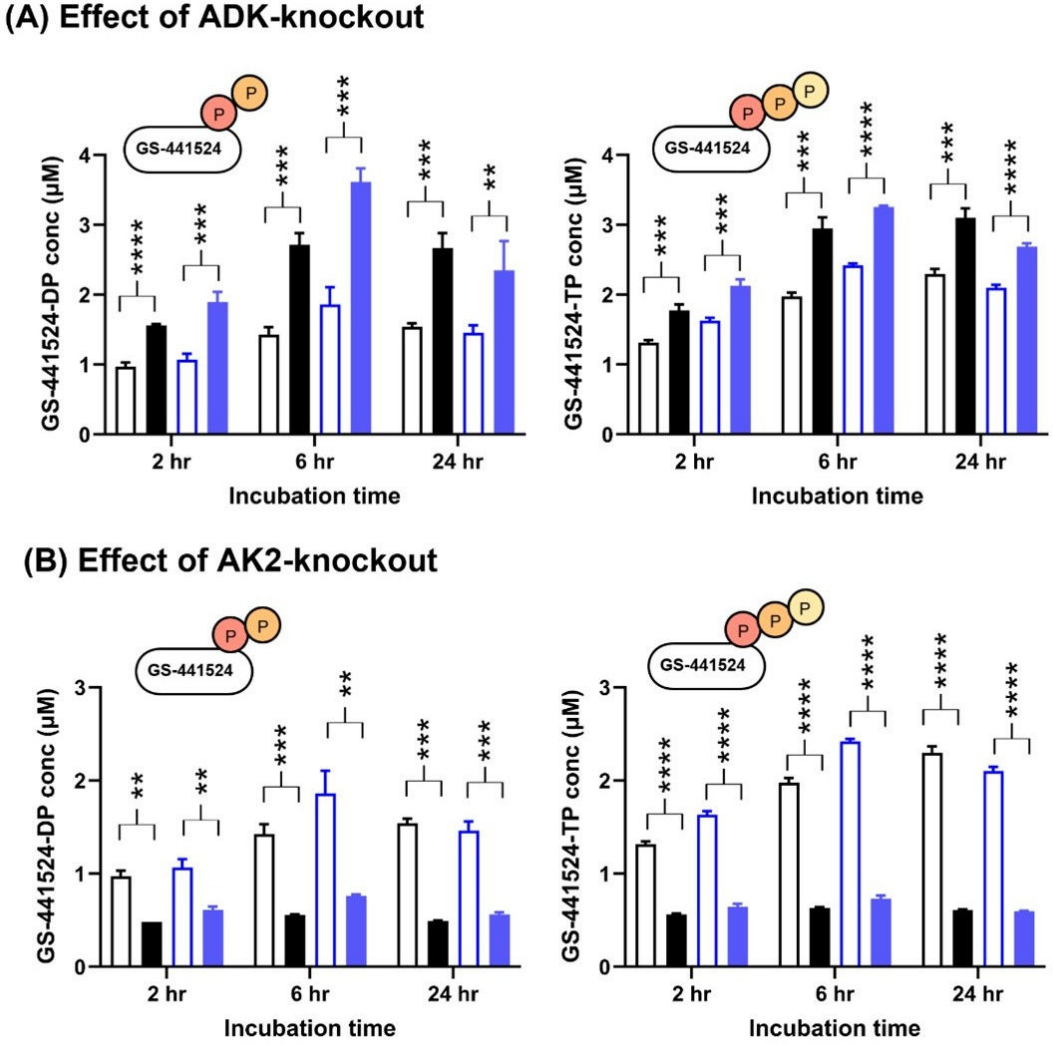
Activation of GS-441524 (black line) and ODV (blue line) in WT (open line), ADK-KO (closed line), and AK2-KO (closed line) of A549-hACE2 cells as a function of time. Data represent an average of three replicates. Asterisks denote *P* values from a multiple unpaired t test (*P < 0.05, **P < 0.01, ***P < 0.001, and ****P < 0.0001). Multiple comparison correction with the Holm-Šídák method using an alpha factor of 0.05 was also performed. (**A**) Effect of ADK-KO on GS-441524-DP and -TP formation after 2, 6, and 24-h incubation of 10 µM GS-441524 or ODV in A549-hACE2 cells. WT + GS-441524 (open black line), ADK-KO + GS-441524 (closed black line), WT + ODV (open blue line), and ADK-KO + ODV (closed blue line). (**B**) Effect of AK2-KO on GS-441524-DP and -TP formation after 2, 6, and 24-h incubation of 10 µM GS-441524 or ODV in A549-hACE2 cells. WT + GS-441524 (open black line), AK2-KO + GS-441524 (closed black line), WT + ODV (open blue line), and AK2-KO + ODV (closed blue line).

To identify the key kinases involved in ODV phosphorylation and their subsequent activity, a total of 11 kinases were selected, and their genes were individually knocked out in A549-hACE cells using CRISPR-Cas9 technology (Table 3 and Materials and Methods; section Creation of Single-Gene Knockout Using A549-hACE2 Cells and Single-Clone Selection). Knockout (KO) cell lines were confirmed by genotyping and protein level using western blot (an example shown in Fig. 8). These KO cells were then evaluated in a metabolism study by measuring metabolites after treating wild-type (WT) and KO cells with ODV or GS-441524 for 2-, 6-, and 24 hours. As shown in Table 4 and Fig. 4, the diphosphate and triphosphate metabolite levels were significantly lower (*P*-values from <0.0001 to <0.05) at all time points in AK2-KO cells and reached a maximal fourfold decrease in metabolite formation, suggesting AK2 is a critical intracellular enzyme to phosphorylate ODV intracellularly. Metabolite levels in ADK-KO cells were moderately elevated (1.3- to 1.9-fold) compared to WT cells, but this increase was consistently observed across all time points in both GS-441524 and ODV-treated cells and was statistically significant. This finding directly contrasts with the conventional and fast-held belief that adenosine kinase, a highly specific phosphotransferase for adenosine, is the most likely enzyme responsible for the conversion of GS-441524 to GS-441524-MP.

**TABLE 3:** Summary of CRISPR-Cas9 knockout studies in A549-hACE2

Enzyme^*a*^	Gene symbol^*b*^	Dependency^*c*^ (in A549 cells)	KO confirmed by Western or Sanger seq^*d*^	Affect cellular metabolite levels?
Adenosine kinase	ADK	0.03152	Yes	Increased
Adenylate kinases	AK1	0.23263	Yes	No
	AK2	0.51623	Yes	Decreased
	AK3	0.01923	Yes	No
	AK4	0.79560	Yes	No
	AK5	0.06315	Yes	No
NDP kinase/NDPK/nucleoside diphosphate kinases	NME1	0.09030	Yes	No
	NME4	0.08112	Yes	No
	NME6	0.61289	Yes	No
	NME7	0.01742	Yes	No
Pyruvate kinase	PKLR	0.08331	Yes	No

^*a*^ The genes in the table showed protein expression in A549 cells based on the proteomic study “Quantitative Proteomics of the Cancer Cell Line Encyclopedia” by Nusinow et al. Cell (2019).

^*b*^ Genes not listed in this table failed the knockout approach due to high essential scores, which are AK6, NME2, PGK1, and PKM. Genes that were not expressed in parental A549 cells are AK7, AK8, and AK9.

^*c*^ Dependency is the concept that a gene is essential for cellular survival or proliferation. The dependency score of a gene is a value between 0 and 1; the higher the value, the higher the impact of the knockout of a particular gene on cell viability. The dependency scores listed here are extracted from the depmap portal (Cancer Dependency Map) https://depmap.org/portal.

^*d*^ KO efficiency of ≥95% was confirmed by Western blot or Sanger sequencing.

**TABLE 4:** Effect of ADK-KO and AK2-KO on the activation of GS-441524 and ODV in A549-hACE2 cells as a function of time

Metabolite measured	Compound	Time	Conc. (µM) WT^*a*^	Conc. (µM) ADK-KO^*a*^	Fold change^*b*^	Conc. (µM) AK2-KO^*a*^	Fold change
GS-441524	GS-441524	2 h	12.05 ± 0.75	15.20 ± 0.42	1.26 ± 0.09	9.62 ± 0.59	0.80 ± 0.07
		6 h	11.89 ± 0.78	13.73 ± 1.20	1.15 ± 0.13	9.94 ± 0.55	0.84 ± 0.07
		24 h	9.67 ± 0.88	10.14 ± 1.11	1.05 ± 0.15	7.06 ± 0.46	0.73 ± 0.08
	ODV	2 h	15.46 ± 1.14	15.93 ± 0.27	1.03 ± 0.08	16.81 ± 1.01	1.09 ± 0.10
		6 h	13.95 ± 0.26	15.05 ± 0.57	1.08 ± 0.05	14.70 ± 0.22	1.05 ± 0.03
		24 h	7.54 ± 0.25	7.70 ± 0.55	1.02 ± 0.08	7.20 ± 0.43	0.95 ± 0.07
GS-441524-MP	GS-441524	2 h	BLQ^*c*^	BLQ	NA^*d*^	BLQ	NA
		6 h	BLQ	BLQ	NA	BLQ	NA
		24 h	BLQ	BLQ	NA	BLQ	NA
	ODV	2 h	BLQ	BLQ	NA	BLQ	NA
		6 h	BLQ	BLQ	NA	BLQ	NA
		24 h	BLQ	BLQ	NA	BLQ	NA
GS-441524-DP	GS-441524	2 h	0.97 ± 0.06	1.57 ± 0.02	1.62 ± 0.10	0.48 ± 0.00	0.49 ± 0.03
		6 h	1.43 ± 0.11	2.71 ± 0.17	1.90 ± 0.18	0.56 ± 0.01	0.39 ± 0.03
		24 h	1.54 ± 0.05	2.67 ± 0.22	1.73 ± 0.15	0.49 ± 0.01	0.32 ± 0.01
	ODV	2 h	1.07 ± 0.09	1.90 ± 0.15	1.78 ± 0.20	0.61 ± 0.04	0.58 ± 0.06
		6 h	1.86 ± 0.25	3.61 ± 0.20	1.94 ± 0.28	0.76 ± 0.02	0.41 ± 0.05
		24 h	1.46 ± 0.10	2.35 ± 0.42	1.61 ± 0.30	0.56 ± 0.02	0.39 ± 0.03
GS-441524-TP	GS-441524	2 h	1.32 ± 0.03	1.78 ± 0.09	1.35 ± 0.07	0.56 ± 0.01	0.43 ± 0.01
		6 h	1.98 ± 0.05	2.95 ± 0.16	1.49 ± 0.09	0.63 ± 0.01	0.32 ± 0.01
		24 h	2.30 ± 0.07	3.10 ± 0.13	1.35 ± 0.07	0.61 ± 0.01	0.26 ± 0.01
	ODV	2 h	1.63 ± 0.04	2.13 ± 0.09	1.31 ± 0.06	0.65 ± 0.03	0.40 ± 0.05
		6 h	2.42 ± 0.03	3.26 ± 0.02	1.35 ± 0.02	0.73 ± 0.03	0.30 ± 0.04
		24 h	2.10 ± 0.05	2.69 ± 0.04	1.28 ± 0.03	0.60 ± 0.01	0.28 ± 0.02

^*a*^ The values reported are average ±standard deviation (SD) from three measurements.

^*b*^ Fold change = KO/WT, the propagated error in SD was calculated using the equation $\sqrt{\begin{array}{c} {\left(\frac{SDwt}{WT}\right)}^{2}+{\left(\frac{SDko}{KO}\right)}^{2} \end{array}}$x Average.

^*c*^ BLQ = below the limit of quantitation (<0.13 µM).

^*d*^ NA = not applicable.

### Effect of ADK- or AK2-KO on the anti-SARS-CoV-2 activity and cytotoxicity of ODV and GS-441524 in A549-hACE2 cells

**Fig 5:**
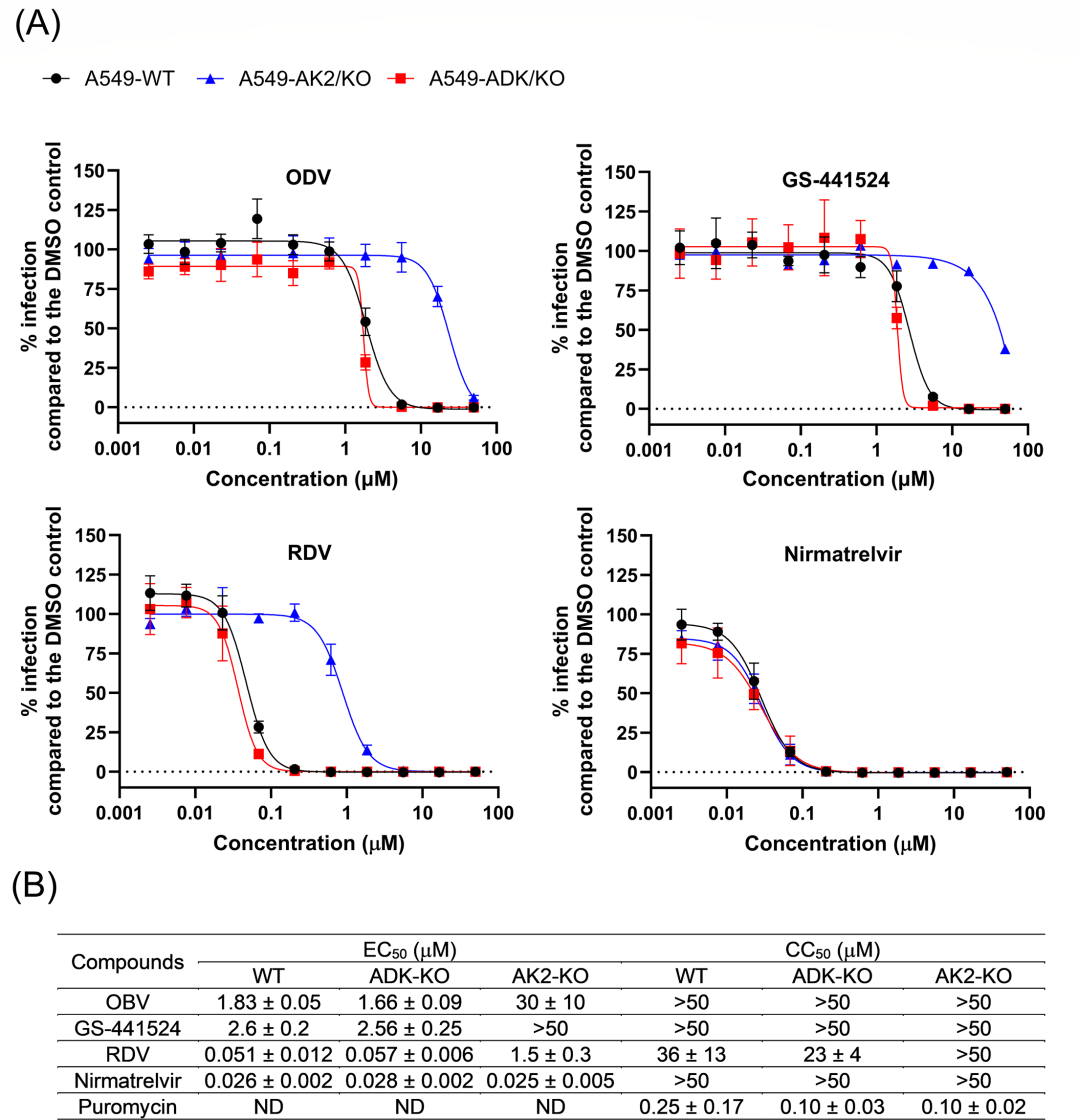
Effect of single-gene knockout on the anti-SARS-CoV-2 activity and cytotoxicity of ODV, GS-441524, and RDV in A549-hACE2 cells after 48-h incubation and summary table. Data represent three independent replicates. (**A**) Compared to the WT cells (black), AK2-KO (blue) resulted in decreased (right-shifted) anti-SARS-CoV-2 activity for the nucleoside/nucleotide analogs ODV, GS-441524, and RDV, but had no effect on nirmatrelvir, a potent and selective inhibitor of SARS-CoV-2 3C-like protease (Mpro). In contrast, ADK-KO (red) showed a minimal effect on compound activity (dose-response curves and EC_50_). (**B**) A summary for the EC_50_ and CC_50_ values of all compounds tested in the WT, ADK-KO, and AK2-KO cells. Data were averages ± SD of four independent experiments.

To further evaluate the effects of altered intracellular metabolism by ADK or AK2, a panel of compounds was tested for antiviral activities against SARS-CoV-2 on A549-hACE2 WT, ADK-KO, or AK2-KO cells. The cells were treated with compounds for 2 days, in the presence or absence of SARS-CoV-2, and the level of SARS-CoV-2 virus was measured using a Nano-Luc (Nluc) reporter assay. Moreover, cytotoxicity was monitored by measuring the cellular ATP levels using a CellTiter-Glo assay. As shown in Fig. 5A, AK2-KO significantly right-shifted the dose-response curves, thus decreasing anti-SARS-CoV-2 activities of the parent nucleoside GS-441524, its 5′-ester prodrug ODV, and its monophosphoramidate prodrug RDV. On the other hand, ADK-KO cells exhibited similar anti-SARS-CoV-2 activities to those of WT cells. The data confirmed that AK2 is a key enzyme in the nucleoside/nucleotide activation pathway. As expected, the antiviral activity of nirmatrelvir, a protease inhibitor that does not require metabolic activation, was not affected by any of the KOs.

### Identification of potential phosphotransferases for the transformation of GS-441524 to GS-441524-MP in ADK-KO cells

Three lines of evidence have thus far suggested that ADK is not the main enzyme responsible for the phosphorylation of GS-441524 to GS-441524-MP: (i) Biochemically, GS-441524 is a poor substrate for human recombinant ADK (Table 2); (ii) in ADK-KO cells, the levels of GS-441524-DP or -TP were increased up to 1.9-fold higher than that of WT cells (Fig. 4); (iii) also in ADK-KO cells, the antiviral EC_50_ values of ODV or GS-441524 were unchanged compared to WT (Fig. 5). One of the explanations is that there is a compensatory enzyme of ADK activities in WT cells, which is then further turned on in ADK-KO cells to compensate. To test this hypothesis, RNA-seq analysis was performed to examine the effect of ADK single-gene knockout in A549-hACE2 cells.

**Fig 6:**
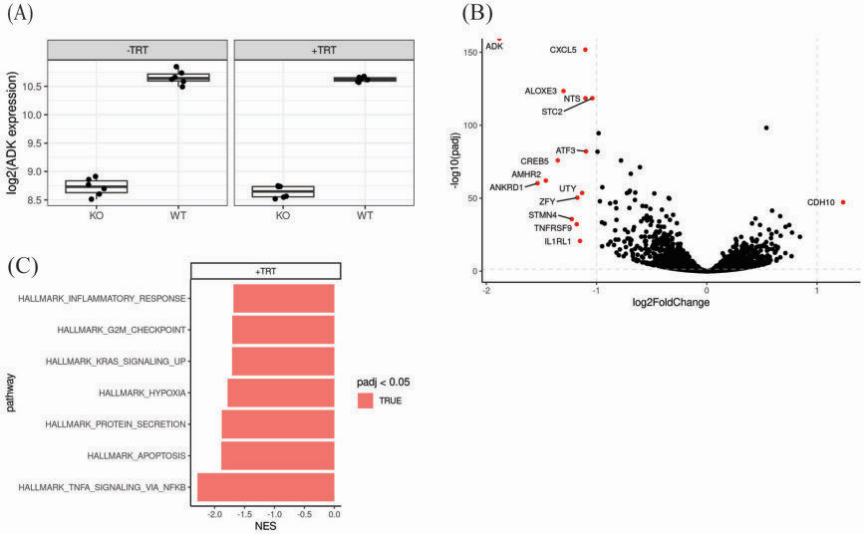
Transcriptional signature associated with ADK knockout. (**A**) ADK normalized expression for DMSO and ODV in ADK knockout (KO) and WT cells. (**B**) Volcano plot showing log2 expression changes and significance values. Red-highlighted points represent genes with adjusted *P*-value < 0.05 and |log2FoldChange| > 1. (**C**) Gene set enrichment analysis (GSEA) on expression changes associated with ODV-treated KO versus WT cells. Values represent normalized enrichment scores from GSEA.

RNA-seq analysis was performed on the WT and ADK-KO cells treated with ODV and GS-441524 for 2, 6, and 24 hours. Data analysis in Fig. 6 was done using samples from a 24-h treatment. RNA-seq data confirmed a significant reduction of ADK expression in ADK-KO cells compared to WT (Benjamini-Hochberg adjusted *P*-value < 0.001; 3.2-fold reduction; Fig. 6A). A total of 15 differentially expressed genes were identified, most of which were downregulated relative to the WT control (14/15 downregulated; Fig. 6B). Cadherin-10 (CDH10) was the only gene observed to be significantly upregulated (Benjamini-Hochberg adjusted *P*-value < 0.001, 2.2-fold increase relative to WT). Gene set enrichment analysis revealed that the downregulated genes were those only associated with inflammatory pathways. However, none of them were previously identified as phosphotransferases (Fig. 6C). In summary, the RNA-seq analysis of WT and ADK-KO cells did not reveal any compensating enzymes that could potentially phosphorylate GS-441524 to its 5′-monophosphate.

## DISCUSSION

In this paper, we elucidated the potential enzymes involved in the activation of GS-441524 and its prodrug ODV, an orally active anti-SARS-CoV-2 nucleoside analog prodrug that has completed Phase III clinical trials for COVID-19 and a Phase III clinical trial for RSV. Our data suggested that CES1, CES2, AK2, and NDP kinases (NM23H1 and NM23H2), phosphoglycerate kinase, and pyruvate kinase (PK) are likely involved in the activation pathway. Most of these enzymes are ubiquitously expressed in human tissues, including blood and lung tissues/cells with high response to SARS-CoV-2 infection (25). CES1 is highly expressed in the liver and adipocytes, while CES2 is mainly expressed in the small intestine and colon (26). Since ODV is administered orally, it is highly feasible that the majority of ODV is metabolized to GS-441524 by the intestinal CES2 and any residual ODV that enters the portal vein is metabolized to GS-441524 in the liver. Our enzymatic study data are highly consistent with the notion that ODV is cleaved pre-systemically based on prior *in vitro* and *in vivo* pharmacokinetic studies (7, 16).

Our study showed that AK2 is a key enzyme involved in GS-441524-MP activation in a well-established model of lung cells (A549 overexpressed with human ACE2), and this is in agreement with a work by Akinci et al. on the key genes involved in RDV activation and cytotoxicity, which was done in liver HepG2 and intestinal HT29 cells (27). A potential limitation of our work is that the findings are only based on two cell lines, the A549-hACE2 cell line and the HeLa cell line, known for its susceptibility to SARS-CoV-2 and RSV infection, respectively; however, they may not fully represent the diverse cell- and tissue-based enzyme expression and activity patterns *in vivo*.

Adenosine plays critical roles in cellular energy homeostasis through multiple adenosine-receptor-dependent and -independent pathways. Its cellular concentration is tightly regulated by several biochemical processes, including the conversion to AMP by ADK and conversion to inosine by adenosine deaminase (28). ADK has two isozymes as alternative splice variants, ADK-long (ADKL) and ADK-short (ADKS), which are identical in sequence and biochemical properties except that ADKL is 21 amino acids longer than ADKs (28). Even though GS-441524 is a *C*-nucleoside with a modified adenine base, its active TP metabolite has been shown to be an excellent competitive substrate for ATP with high incorporation efficiency during SARS-CoV-2 RdRp-catalyzed RNA synthesis (9). As well as other viral RNA polymerases (29). Phosphorylation of GS-441524 to the MP metabolite has been hypothesized to be catalyzed primarily by human adenosine kinase, which is also based on our collective knowledge of the substrate specificity of a wide range of phosphotransferases and their involvement in the activation of anti-cancer and antiviral nucleoside/tide analogs since the 1950s (30, 31). However, multiple lines of evidence from our work showed that this analog is a poor substrate (>1,000,000-fold less efficient) for ADK compared to that of the natural substrate adenosine, suggesting an alternative enzyme(s) is involved in the formation of GS-441524-MP. This hypothesis is further supported by three studies in ADK-KO cells: (i) ADK-KO showed increased GS-441524-DP and TP formation, supporting the presence of an alternative pathway; (ii) ADK-KO caused no impact on the anti-SARS-CoV-2 activities of ODV and GS-441524; and (iii) RNA-seq study of ADK-KO cells showed significant downregulation of genes involved in inflammation in TNF alpha pathways, consistent with the reports on adenosine receptor-mediated inflammation (31). However, we were not able to identify an upregulated compensatory phosphotransferase or a downregulated AMP phosphatase. Future studies are needed to identify enzymes responsible for activating GS-441524 to its MP metabolite.

**Fig 7:**
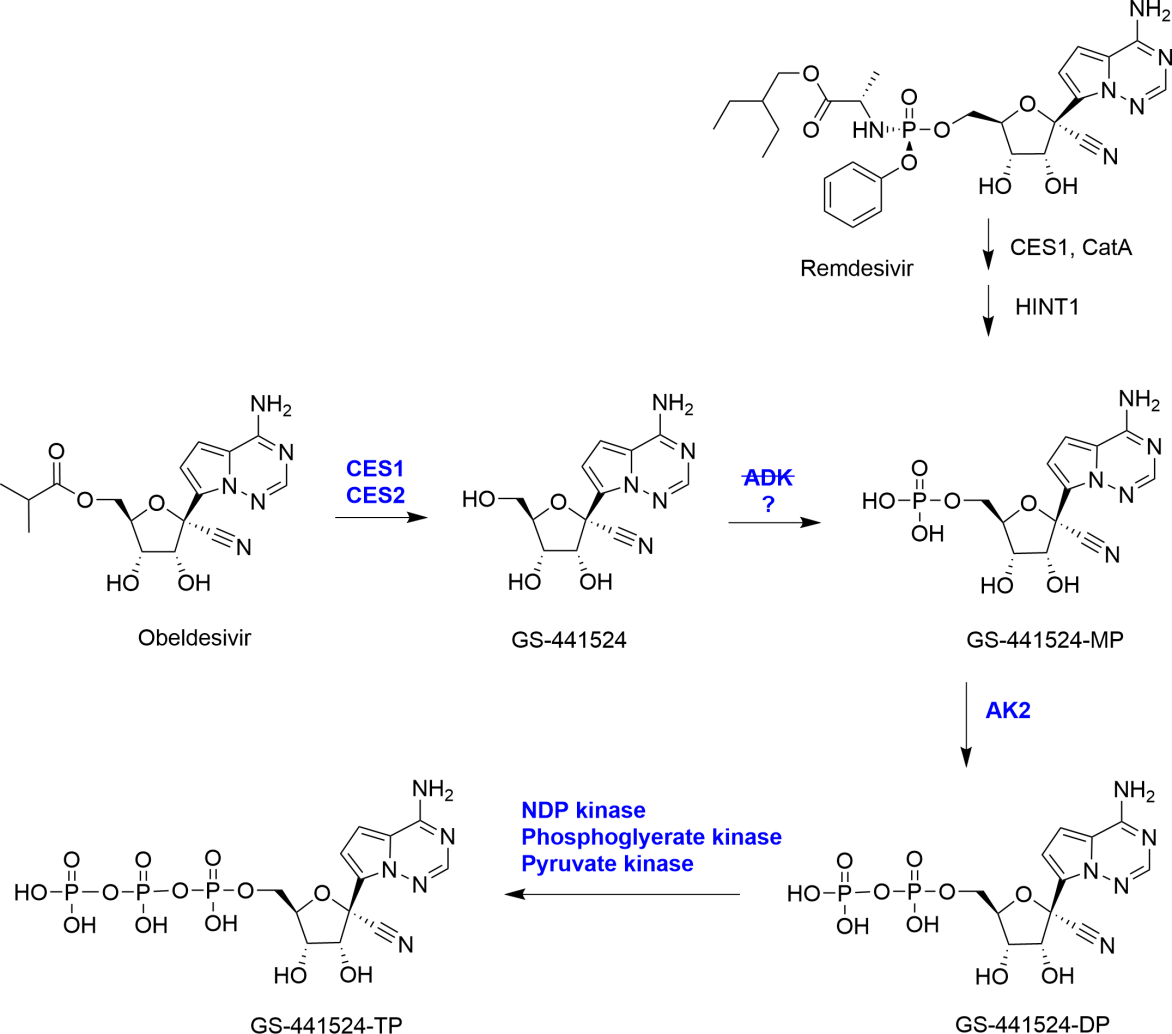
Summary of obeldesivir and remdesivir activation pathway. The major metabolic enzymes identified or ruled out in this study are highlighted in blue.

In conclusion, using both biochemical and cell-based assays, we identified several key enzymes involved in the activation pathway of ODV and GS-441524 and completed the previously reported profiling of the RDV activation pathway that we initiated in 2021 (25) (Fig. 7). AK2 was identified as a rate-limiting enzyme in the activation of GS-441524-MP to DP, a metabolic step to the active triphosphate metabolite formed by ODV and RDV. The observed low catalytic efficiency of ADK towards GS-441524 is both surprising and intriguing, and it warrants further research into this critical step in the activation pathway of ODV.

## MATERIALS AND METHODS

### Reagents

All enzymes used are human recombinant enzymes. Human dGK was purified by Dr. Liya Wang at SLU—Swedish University of Agricultural Sciences. Commercial enzyme sources were described in the assay methods.

The 5′ NT gene encoding amino acid M1-E561 was cloned into a pET24b vector in frame with a C-terminal 6x-His tag. Expression of the His-tagged fusion protein was achieved by transforming the plasmid into BL21 (DE3) *E. coli* cells and inducing culture in LB medium with IPTG to a final concentration of 0.5 mM at 15°C for 16 hours. Induced culture was harvested and stored at −80°C. Protein purification was carried out as described previously with modifications (24). The cell pellet was resuspended in lysis buffer (20 mM Tris-HCl, pH 8.0, 20 mM NaCl, 1 mM EGTA, 1 mM β-ME, 0.1 mM PMSF) supplemented with EDTA-free protease inhibitor (Roche). The cell suspension was passed through a microfluidics high-pressure homogenizer, and the lysate was centrifuged at 16,000 rpm for 30 minutes at 4°C. The supernatant was loaded onto a Hi-Trap Talon crude column (Cytiva, Marlborough, MA) to enrich the target protein. The column was washed with buffer A (20 mM Tris-HCl [pH 8.0], 600 mM NaCl, 5% glycerol), and the target protein was eluted using buffer B (20 mM Tris [pH 8.0], 600 mM NaCl, 5% glycerol, 300 mM imidazole). The sample was buffer exchanged and loaded over a Mono Q 10/100 GL column equilibrated with buffer C (20 mM Tris-HCl [pH 7.5], 150 mM NaCl, 5% glycerol). The target protein was eluted using a linear gradient using buffer D (20 mM Tris-HCl [pH 7.5], 1 M NaCl, 5% glycerol). The major peak from the elution was loaded over a Superdex 200 Increase 10/300 GL column (Cytiva) equilibrated in storage buffer (10 mM Tris-HCl [pH 7.5], 200 mM KCl, 10% glycerol). Two peaks from elution were collected individually, and quality control experiments were performed separately. The A260/A280 of the fractions from both peaks was calculated to be <0.6, suggesting no nucleic acid contamination. Analytical SEC-HPLC quality control analysis of the purified preps indicated an elution pattern of the protein corresponding to ~300 kDa. Intact LC-MS and peptide mapping analysis were used to verify the molecular weight (MW) and identity of the proteins.

All sgRNAs for single-gene CRISPR knockout were ordered from Synthego (Redwood City, CA 94063). Refer to section Creation of Single-Gene Knockout Using A549-hACE2 Cells and Single-Clone Selection for sgRNA information.

### Cell culture

A549-human angiotensin converting enzyme 2 (hACE2) human epithelial lung cells were licensed from the University of Texas Medical Branch (UTMB) (32). The cells were cultured in DMEM media (Thermo Fisher/Gibco, cat# 11995073), supplemented with 10% FBS (Hyclone SH30071-03), 1× Pen-Strep-Glutamine (Thermo Fisher/Gibco, cat#10378016), and Blasticidin (10 µg/mL) (Thermo Fisher, cat#A1113903). Cells were passaged twice a week and used for experiments at passages 5–20.

### Biochemical assays

#### Conversion of parent nucleoside GS-441524 to GS-441524-MP

##### Adenosine kinase assay

Phosphorylation of adenosine and GS-441524 by adenosine kinase (ADK, ACROS, Catalog# 58-61-7, Lot#A0417098) was performed in reactions (20 mL) containing 10 nM ADK with 20 µM ATP (phosphate donor) in 50 mM Tris-HCl (pH 7.5), 2 mM MgCl_2_, 125 mM KCl, 2 mM DTT, and 0.5 mg/mL BSA. The final concentration of guanosine and GS-441524 was 200 mM (the highest starting concentration) with twofold dilutions. Reactions were incubated for 60 minutes at room temperature. Activity of guanosine-inosine kinase (GSK) was measured by the ADP-Glo Kinase Assay Kit (Promega). Briefly, 20 mL ADP-Glo Reagent was added to terminate the reaction and deplete remaining ATP. The mixture was incubated for 40 minutes at room temperature. 40 mL of Kinase Detection Reagent was then added to convert ADP to ATP for 30 minutes at room temperature. The newly synthesized ATP was measured using a luciferase/luciferin reaction. Luminescence (RLU) was read using a Molecular Devices ID5 plate reader. RLU values were plotted against the substrate concentration, and *V*_max_ and *K*_m_ were calculated by nonlinear regression using GraphPad Prism (Version 8, La Jolla, CA).

##### Guanosine-inosine kinase assay

Phosphorylation of guanosine and GS-441524 by GSK (MyBiosource, Catalog# MBS1122404, Lot# DD04957a7g0) was performed in reactions (20 mL) containing 500 nM GSK with 1 mM ATP (phosphate donor) in 100 mM Tris-HCl (pH 7.5) and 100 mM KCl. The final concentration of guanosine and ODV was 100 mM (the highest starting concentration) with twofold dilutions. Reactions were incubated for 60 minutes at room temperature. Activity of GSK was measured by ADP-Glo Kinase Assay Kit (Promega). Briefly, 20 mL ADP-Glo Reagent with 10 mM MgCl_2_ was added to terminate the reaction and deplete remaining ATP. The mixture was incubated for 40 minutes at room temperature. 40 mL of Kinase Detection Reagent was then added to convert ADP to ATP for 40 minutes at room temperature. The newly synthesized ATP was measured using a luciferase/luciferin reaction. Relative luminescence units (RLUs) were read using a Molecular Devices ID5 plate reader. RLU values were plotted against the substrate concentration, and *V*_max_ and *K*_m_ were calculated by nonlinear regression using GraphPad Prism (Version 8, La Jolla, CA).

##### Deoxyguanosine kinase assay

Phosphorylation of deoxyguanosine and GS-441524 by deoxyguanosine kinase (dGK) was performed in reactions (20 mL) containing 250 nM dGK with 1,000 mM ATP (phosphate donor) in 50 mM Tris-HCl (pH 7.5), 100 mM KCl, and 1 mM MgCl_2_. The final concentrations of deoxyguanosine and GS-441524 were 100 mM and 400 mM (the highest starting concentration), respectively, with twofold dilutions. Reactions were incubated for various time points at room temperature. Activity of dGK was measured by ADP-Glo Kinase Assay Kit (Promega). Briefly, 20 mL ADP-Glo Reagent with 10 mM MgCl_2_ was added to terminate the reaction and deplete remaining ATP. The mixture was incubated for 40 minutes at room temperature. 40 mL of Kinase Detection Reagent was then added to convert ADP to ATP for 40 minutes at room temperature. The newly synthesized ATP was measured using a luciferase/luciferin reaction. Luminescence was read using a Molecular Devices ID5 plate reader. Rates were determined and plotted against the substrate concentration. *V*_max_ and *K*_m_ were calculated by nonlinear regression using GraphPad Prism (Version 8, La Jolla, CA).

##### Deoxycytidine kinase assay

Phosphorylation of deoxycytidine and GS-441524 by dCK (Prosci, Catalog# 92-064) was performed in reactions (20 µL) containing 3.4 µM dCK with 40 µM ATP (phosphate donor) in 50 mM Tris-HCl (pH 7.5), 2 mM MgCl_2_, 125 mM KCl, 10 mM DTT, and 0.5 mg/mL BSA. The final concentration of guanosine and GS-441524 was 100 µM (the highest starting concentration) with twofold dilutions. Reactions were incubated for 60 minutes at room temperature. Activity of dCK was measured by the ADP-Glo Kinase Assay Kit (Promega). Briefly, 20 µL ADP-Glo Reagent was added to terminate the reaction and deplete remaining ATP. The mixture was incubated for 40 minutes at room temperature. 40 µL of Kinase Detection Reagent was then added to convert ADP to ATP for 30 minutes at room temperature. The newly synthesized ATP was measured using a luciferase/luciferin reaction. Luminescence was read using a Molecular Devices ID5 plate reader. RLU values were plotted against the substrate concentration, and *V*_max_ and *K*_m_ were calculated by nonlinear regression using GraphPad Prism (Version 8, La Jolla, CA).

##### Thymidine kinase assay

Phosphorylation of thymidine and GS-441524 by thymidine kinase 1 (TK1, R&D Sciences, Catalog# 8180-TK, Lot# DCYS0221021) and thymidine kinase 2 (TK2, Novus, Catalog# NBP1-98864, Lot# 12166301) was performed in reactions (20 mL) containing TK1 or TK2 with 1 mM ATP (phosphate donor) in 50 mM Tris-HCl (pH 7.5), 100 mM KCl, and 1 mM MgCl_2_. The concentrations of TK1 used for thymidine and GS-441524 were 5 nM and 500 nM, respectively. Thymidine and GS-441524 were tested at 10 mM (the highest starting concentration) with twofold dilutions. The concentration of TK2 used for thymidine and GS-441524 was 250 nM with thymidine at 25 mM and GS-441524 at 100 mM (the highest starting concentration) with twofold dilutions. Reactions were incubated for 60 minutes at room temperature. Activity of TK1 and TK2 was measured by ADP-Glo Kinase Assay Kit (Promega). Briefly, 20 mL ADP-Glo Reagent with 10 mM MgCl_2_ was added to terminate the reaction and deplete remaining ATP. The mixture was incubated for 40 minutes at room temperature. 40 mL of Kinase Detection Reagent was then added to convert ADP to ATP for 40 minutes at room temperature. The newly synthesized ATP was measured using a luciferase/luciferin reaction. Luminescence was read using a Molecular Devices ID5 plate reader. RLU values were plotted against the substrate concentration, and *V*_max_ and *K*_m_ were calculated by nonlinear regression using GraphPad Prism (Version 8, La Jolla, CA).

##### Uridine cytidine kinase assay

Phosphorylation of uridine and GS-441524 by UCK1 (Creative Biomart, Catalog# UCK1-199H, Lot# PSU905217) and UCK2 (Creative Biomart, Catalog# UCK2-30101TH, Lot# PSU105217) was performed in reactions (20 mL) with ATP (phosphate donor) in 50 mM Tris-HCl (pH 7.5), 100 mM KCl, and 0.5 mg/mL BSA. The concentrations of UCK1 used for uridine and ODV were 50 nM and 250 nM, respectively, with uridine at 25 mM and GS-441524 at 100 mM (the highest starting concentration), with twofold dilutions. The concentrations of UCK2 used for uridine and GS-441524 were 2 nM and 50 nM, respectively, with uridine at 1 mM and GS-441524 at 100 mM (the highest starting concentration) with twofold dilutions. Reactions were incubated for 60 minutes at room temperature. Activity of UCK was measured by ADP-Glo Kinase Assay Kit (Promega). Briefly, 20 mL ADP-Glo Reagent with 10 mM MgCl_2_ was added to terminate the reaction and deplete remaining ATP. The mixture was incubated for 40 minutes at room temperature. 40 mL of Kinase Detection Reagent was then added to convert ADP to ATP for 40 minutes at room temperature. The newly synthesized ATP was measured using a luciferase/luciferin reaction. Luminescence was read using a Molecular Devices ID5 plate reader. RLU values were plotted against the substrate concentration, and *V*_max_ and *K*_m_ were calculated by nonlinear regression using GraphPad Prism (Version 8, La Jolla, CA).

##### High *K*_m_ 5′-NT, ectonucleotidase CD73 assay

Phosphorylation of high *K*_m_ 5′-NT and GS-441524 by high *K*_m_ 5′-Nucleotidase (high *K*_m_ 5′-NT, Gilead, Lot#11211-001) was performed in reactions (20 µL) containing 2 µM high *K*_m_ 5′ nucleotidase with 5 mM IMP (phosphate donor) in 50 mM imidazole-HCl (pH 6.5), 20 mM MgCl_2_, 5 mM ATP, and 0.5 mg/mL BSA. The final concentration of guanosine and GS-441524 was 200 µM (the highest starting concentration) with twofold dilutions. Reactions were incubated for 60 minutes at room temperature. To terminate the reaction, 20 µL of acetonitrile was added per reaction. The samples were frozen for further analysis. Metabolism of GS-441524 (monophosphate of GS-441524 and inosine) by high *K*_m_ 5′-NT was measured by LC/MS analysis (as described below).

### Conversion of GS-441524-MP to GS-441524-DP

#### Adenylate kinase (AK1 and AK2) assay

Phosphorylation of ^15^N_5_-adenosine monophosphate by adenylate kinase 1 (AK1, Novus Biologicals, Catalog# NBP1-50855, Lot# 1077901) and adenylate kinase 2 (AK2, Novus Biologicals, Catalog# NBP1-18530, Lot# 2021001) was performed in reactions (125 mL) containing 0.2 nM AK1 and 0.5 nM AK2, respectively, and 5 mM ATP (phosphate donor) in 50 mM Tris-HCl (pH 7.5), 5 mM MgCl_2_, and 100 mM KCl. The final concentrations of ^15^N_5_-adenosine monophosphate were 0, 100, 500, and 1,500 mM. Reactions were incubated for various time points at 37°C. For each time-point, 25 mL aliquots of the reaction were quenched in 225 mL ice-cold 70% MeOH with 2.5 mM Cl-ATP and placed on ice until all reaction data points were collected. Product formation rates were determined and plotted against the substrate concentration. *V*_max_ and *K*_m_ were calculated by nonlinear regression using GraphPad Prism (Version 8, La Jolla, CA).

Phosphorylation of GS-441524-MP by adenylate kinase 1 (AK1, Novus Biologicals, Catalog# NBP1-50855, Lot# 1077901) and adenylate kinase 2 (AK2, Novus Biologicals, Catalog# NBP1-18530, Lot# 2021001) was performed in reactions (125 mL) containing 1.25 nM AK1 and 5 nM AK2, respectively, and 5 mM ATP (phosphate donor) in 50 mM Tris-HCl (pH 7.5), 5 mM MgCl_2_, and 100 mM KCl. The final concentrations of GS-441524-MP were 0, 0.1, 0.5, 2, and 10 mM. Reactions were incubated for various time points at 37°C. For each time point, 25 mL aliquots of the reaction were quenched in 225 mL ice-cold 70% MeOH with 2.5 mM Cl-ATP and placed on ice until all reactions were collected and the samples were analyzed by LC/MS. Rates were determined and plotted against the substrate concentration. *V*_max_ and *K*_m_ were calculated by nonlinear regression using GraphPad Prism (Version 8, La Jolla, CA).

#### Guanylate kinase assay

Phosphorylation of guanosine monophosphate and GS-441524-MP (GS-719700) by guanylate kinase (GMK, R&D Sciences, Catalog# 9267-GU) was performed in reactions (20 mL) containing 0.3 nM GMK and 100 mM ATP (phosphate donor) in 25 mM HEPES (pH 7.0), 150 mM NaCl, 10 mM MgCl_2_, and 10 mM CaCl_2_. The final concentration of guanosine monophosphate and GS-441524-MP was 250 mM (the highest starting concentration) with twofold dilutions. Reactions were incubated for various time points at room temperature. Activity of GMK was measured by ADP-Glo Kinase Assay Kit (Promega). Briefly, 20 mL ADP-Glo Reagent with 10 mM MgCl_2_ was added to terminate the reaction and deplete remaining ATP. The mixture was incubated for 40 minutes at room temperature. 40 mL of Kinase Detection Reagent was then added to convert ADP to ATP for 40 minutes at room temperature. The newly synthesized ATP was measured using a luciferase/luciferin reaction. Luminescence was read using a Molecular Devices ID5 plate reader. Rates were determined and plotted against the substrate concentration. *V*_max_ and *K*_m_ were calculated by nonlinear regression using GraphPad Prism (Version 8, La Jolla, CA).

#### Thymidylate kinase assay

Phosphorylation of thymidine monophosphate and GS-441524-MP by thymidylate kinase (TMK, Creative Biomart, Catalog# DTYMK-3562H) was performed in reactions (20 mL) containing 500 nM TMK and 1 mM ATP (phosphate donor) in 50 mM Tris-HCl (pH 7.5), 1 mM MgCl_2_, and 100 mM KCl. The final concentration of thymidine monophosphate and GS-441524-MP was 200 mM (the highest starting concentration) with twofold dilutions. Reactions were incubated for various time points at room temperature. Activity of TMK was measured by ADP-Glo Kinase Assay Kit (Promega). Briefly, 20 mL ADP-Glo Reagent with 10 mM MgCl_2_ was added to terminate the reaction and deplete remaining ATP. The mixture was incubated for 40 minutes at room temperature. 40 mL of Kinase Detection Reagent was then added to convert ADP to ATP for 40 minutes at room temperature. The newly synthesized ATP was measured using a luciferase/luciferin reaction. Luminescence was read using a Molecular Devices ID5 plate reader. Rates were determined and plotted against the substrate concentration. *V*_max_ and *K*_m_ were calculated by nonlinear regression using GraphPad Prism (Version 8, La Jolla, CA).

#### Cytidine monophosphate kinase assay (uridine monophosphate/cytidine monophosphate kinase)

Phosphorylation of AMP and GS-441524-MP by cytidine monophosphate kinase (CMK, Novus Biologicals, Catalog# NBPI-51052, Lot# 22101301) was performed in reactions (20 mL) containing 200 nM CMK and 1 mM ATP (phosphate donor) in 50 mM Tris-HCl (pH 7.5), 1 mM MgCl_2_, and 50 mM KCl. The final concentrations of uridine monophosphate and GS-441524-MP were 5 mM and 4.475 mM (the highest starting concentration), respectively, with twofold dilutions. Reactions were incubated for 60 minutes at room temperature. The activity of CMK was measured by ADP-Glo Kinase Assay Kit (Promega). Briefly, 20 mL ADP-Glo Reagent with 10 mM MgCl_2_ was added to terminate the reaction and deplete remaining ATP. The mixture was incubated for 40 minutes at room temperature. 40 mL of Kinase Detection Reagent was then added to convert ADP to ATP for 40 minutes at room temperature. The newly synthesized ATP was measured using a luciferase/luciferin reaction. Luminescence was read using a Molecular Devices ID5 plate reader. RLU values were plotted against the substrate concentration, and *V*_max_ and *K*_m_ were calculated by nonlinear regression using GraphPad Prism (Version 8, La Jolla, CA).

### Conversion of GS-441524-DP to GS-441524-TP

#### Creatine kinase assay

Phosphorylation of ADP and GS-441524-DP by creatine kinase MM (CKMM, R&D Systems, Catalog# 9071-CK, Lot# DFKC0121081) and creatine kinase MB (CKMB, Sigma, Catalog# C0984, Lot# SLCN0124) was performed in reactions (50 mL) containing 2.5 nM CKMM or 10 nM CKMB and 4 mM creatine phosphate (phosphate donor) in 25 mM HEPES (pH 7.0), 150 mM NaCl, 10 mM MgCl_2_, and 10 mM CaCl_2_. The final concentration of ADP and GS-441524-DP was 1 mM (the highest starting concentration) with twofold dilutions. Reactions were incubated for 60 minutes at room temperature. Activity of CK was measured by Kinase-Glo Max Assay Kit (Promega). Briefly, 50 mL of Kinase-Glo Max reagent was added to the 50 mL reaction. The mixture was incubated for 10 minutes at room temperature. Luminescence was read using a Molecular Devices ID5 plate reader. RLU values were plotted against the substrate concentration and *V*_max_ and *K*_m_ were calculated by nonlinear regression using GraphPad Prism (Version 8, La Jolla, CA).

#### Pyruvate kinase assay

Phosphorylation of ADP and GS-441524-DP by pyruvate kinase LR (PKLR, R&D Systems, Catalog# 8659-PK, Lot# DEDZ0121111) and pyruvate kinase M2 (PKM2, R&D Systems, Catalog# 7244-PK, Lot# DCFE0521121) was performed in reactions (50 mL) containing 1.25 nM PKLR or 1.0 nM PKM2 and 1.25 mM phosphoenol pyruvate (phosphate donor) in 100 mM MES (pH 6.5), 5 mM MgCl_2_, and 20 mM KCl. The final concentration of ADP and GS-5425-DP was 1250 mM (the highest starting concentration) with twofold dilutions. Reactions were incubated for various time points at room temperature. Activity of PK was measured by Kinase-Glo Max Assay Kit (Promega). Briefly, 50 mL of Kinase-Glo Max reagent was added to the 50 mL reaction. The mixture was incubated for 10 minutes at room temperature. Luminescence was read using a Molecular Devices ID5 plate reader. Rates were determined and plotted against the substrate concentration. *V*_max_ and *K*_m_ were calculated by nonlinear regression using GraphPad Prism (Version 8, La Jolla, CA).

#### Phosphoglycerate kinase assay

Phosphorylation of ADP and GS-441524-DP by PGK (R&D Systems, Catalog# 5455-PK, Lot# SNS0220091) was performed in reactions (50 mL) containing 1.0 nM and 25 nM PGK for ADP and GS-441524-DP, respectively, with 1 mM DL-glyceraldehyde-3-phosphate (GAP, Sigma, Catalog# G5251), 5 ng/mL glyceraldehyde-3-phosphate dehydrogenase (GAPDH, Sigma, Catalog# G5537), 0.3 mM β-nicotinamide adenine dinucleotide (β-NAD, Sigma, Catalog# N6522) in 5 mM KH_2_PO_4_ (pH 7.0), 5 mM MgSO_4_, and 100 mM glycine. The final concentrations of ADP and GS-441524-DP were 200 mM and 500 mM (the highest concentration), respectively, with twofold dilutions. The activity of PGK was measured by the production of NADH. Absorbance (335 nm) was read in kinetic mode for 30 minutes using a Molecular Devices ID5 plate reader. Rates were determined and plotted against the substrate concentration. *V*_max_ and *K*_m_ were calculated by nonlinear regression using GraphPad Prism (Version 8, La Jolla, CA).

### Nucleoside diphosphate kinases

#### (Non-metastatic protein 23, NM23, NDPK-A and NDPK-B)

Phosphorylation of ^15^N_5_-ADP and GS-441524-DP by non-metastatic protein 23 homolog 1 (NM23-H1, R&D Systems, Catalog# 10495-NM-050, Lot# TCO0221121) and non-metastatic protein 23 homolog 2 (NM23-H2, R&D Systems, Catalog# 10466-NM-050, Lot# TAZ0321061) was performed in reactions (125 mL) containing 0.5 nM NM23-H1 or NM23-H2 and 2 mM ATP (phosphate donor) in 50 mM Tris-HCl (pH 7.5) and 100 mM KCl. The final concentrations of ^15^N_5_-adenosine diphosphate were 0, 30, 100, 300, and 900 mM. Reactions were incubated for various time points at 37°C. For each time point, 25 mL aliquots of the reaction were quenched in 225 mL ice-cold 70% MeOH with 2.5 mM Cl-ATP and placed on ice until all reactions were collected. Samples were analyzed by LC-MS/MS.

### Bioanalysis of ^15^N_5_-AMP, ^15^N_5_-ADP, ^15^N_5_-ATP, IMP, GS-441524-MP, GS-441524-DP, and GS-441524-TP

The biochemical assay samples were harvested and extracted with a fivefold volume of extraction buffer (70% methanol containing 250 nM chloro-adenosine triphosphate as the internal standard). All the samples were then filtered using a 0.2 mm 96-well polypropylene filter plate (Varian Captiva). The filtrates were collected into a clean 96-well Eppendorf LoBind plate and diluted with 1 mM ammonium phosphate buffer (pH = 7) for analysis by liquid chromatography-tandem mass spectrometry (LC-MS/MS). The Shimadzu LC-20ADXR ternary pump system (Columbia, MD) was connected to a Sciex Triple Quad 6500 mass spectrometer (Framingham, MA), which was operated in positive ion multiple reaction monitoring (MRM) mode. Analytes were separated by a 2.5 μm 2.0 × 50 mm Luna C18(2) HST column using an ion pairing buffer containing 3 mM ammonium formate (pH 5.0) with 10 mM dimethylhexylamine and a multistage linear gradient from 2.5% to 50% acetonitrile at a flow rate of 0.35 mL/min over 5 minutes. The analyte to internal standard peak area ratios were plotted to determine depletion or formation of the analytes.

### Creation of single-gene knockout using A549-hACE2 cells and single-clone selection

Knockout of genes by CRISPR-Cas9 occurs through indel mutations. A single-guide RNA (sgRNA) guides Cas9 to a precise location in the host genome, where it creates a double-stranded break in the genomic DNA. Cells can survive a double-stranded break when an error-prone repair mechanism like nonhomologous end joining results in the insertion or deletion of one or more base pairs, precluding further binding of the sgRNA. Repairs that result in frameshift mutations can cause an early stop codon in the targeted coding sequence, thereby disrupting gene function, as shown in Fig. 5.

**Fig 8:**
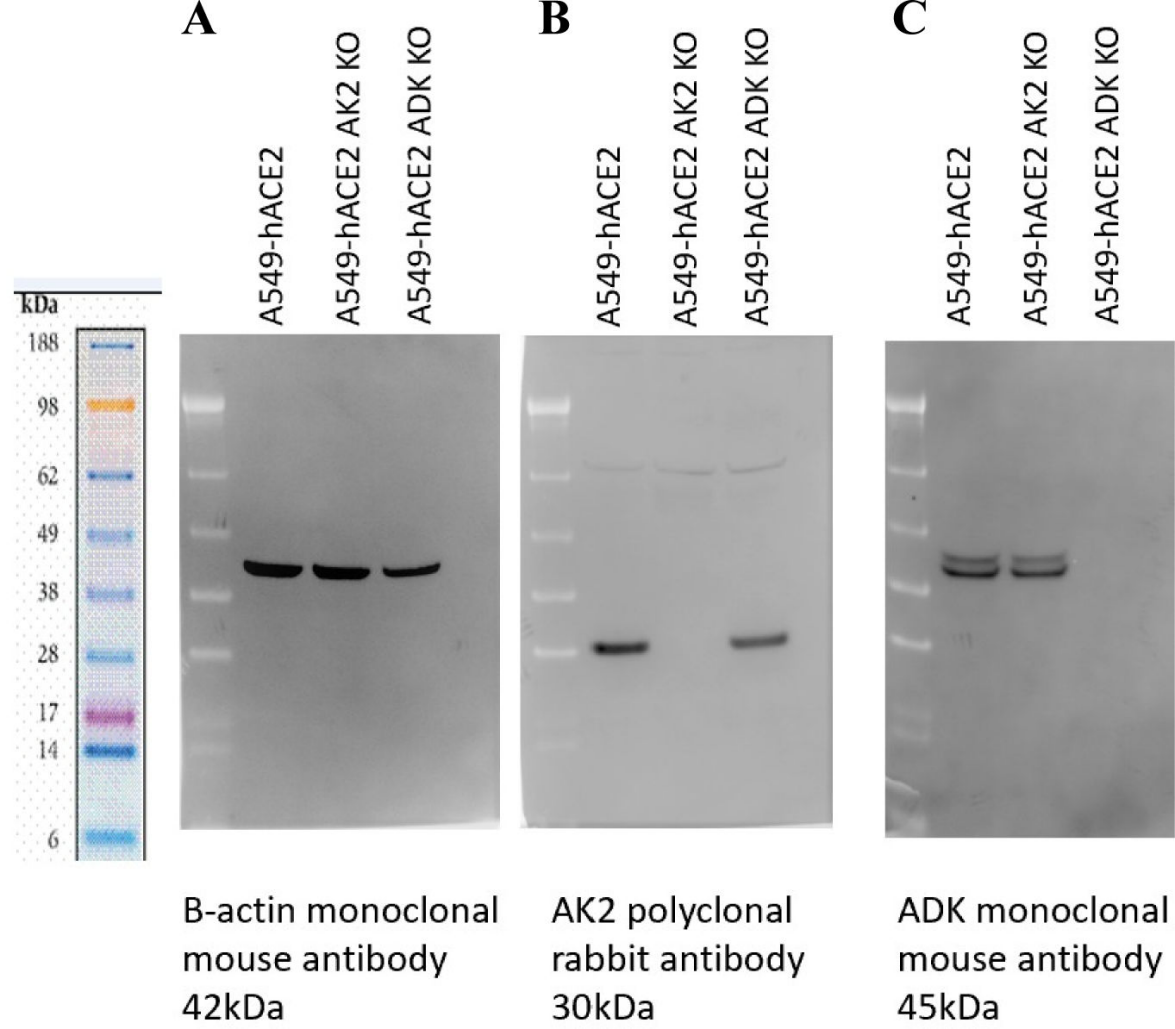
The confirmation of AK2 and ADK gene knockout by western blot. (**A**) β-actin gene showed similar expression levels in A549-hACE2 (wt), A549-hACE2 AK2-KO, and A549-hACE2 ADK-KO cells. (**B**) AK2 gene expression showed a significantly low level in A549-hACE2 AK2-KO cells compared to A549-hACE2 (wt) and A549-hACE2 ADK-KO. (**C**) ADK gene expression showed a significantly low level in A549-hACE2 ADK-KO cells compared to A549-hACE2 (wt) and A549-hACE2 AK2-KO.

Specific gene knockout cells were generated by assembling RNP complexes of 30 pmol sgRNA (Synthego, Table 5) for each targeted gene with 2 μg of Cas9 protein (Thermo, TrueCut Cas9 Protein, cat# A36498) and transducing the complexes into A549-hACE2 cells using the Neon electroporation system (Thermo Fisher) according to the manufacturer’s instructions. The knockout cells were confirmed by PCR amplification (Table 6 for primer information) and Sanger sequencing analysis, or by Western blot using a specific antibody (an example shown in Fig. 8). Single-clone isolation was carried out using limiting dilution cloning in 96-well plates. Refer to the table for sgRNA and PCR primer information, as well as sequencing details.

**TABLE 5:** Information on sgRNA of each knockout gene

Gene	sgRNA 1	sgRNA 2	sgRNA 3
ADK	5′-AAAAAUGUUGCUGCUUUGUG- 3'	5′-CUUCAGCAGCUUUUCUCUUC- 3'	5′-CAUGCAUCACUGGUGACAAC- 3'
AK1	5′-AGGAGAAGAGUUUGAGCGAC- 3'	5′-GCCUUUGGAAGUAUUGACUU- 3'	5′-UGUGCCCCAGGAGACAGUGU- 3'
AK3	5′-ACAUGCUGCGGGGCACAGGU- 3'	5′-UGUAGUGAUGCGCGACGACA- 3'	5′-CGGCCAUGGGGGCGUCCGCG- 3'
AK4	5′-CCGCAGCCCCUCACCGGUGC- 3'	5′-GGACUGGGGCUCCCGGAUCA- 3'	
AK5	5′-CGGAGAUCCUUUCUAAGAAA- 3'	5′-CAAGUAUUCUACUGGAUCUU- 3'	5′-AUGGGAUACAUUUGUAAGCC- 3'
NME1	5′-GGUACGCUCACAGUUGGCCA- 3'	5′-AUCAAGCGUUUUGAGCAGAA- 3'	5′-UACUAUGAAGGAAUCAAAAU- 3'
NME4	5′-CUUUUGAAGGAGGGCCCUCC- 3'	5′-AGCCCGAUGGCGUGCAACGG- 3'	5′-GGCGGGGCUUCACGCUGGUG- 3'
NME6	5′-CUGAGGGCUUCGCAAGAUUG- 3'	5′-GUAGCAAUUCCCAGGGCAUG- 3'	5′-AGUCGCCCAUCCACUGAUUC- 3'
NME7	5′-UAGGACCAGUUGUAAUAAAC- 3'	5′-GGGACCUGCAAACUCUGGAG- 3'	5′-UUAUGCCAUCUGUUCCAAAG- 3'
PKLR	5′-CGUUGGCGAUGGACUCAGCA- 3'	5′-CCGCACUGGGAUCCUGCAGG- 3'	5′-CACGGGCCGGUAGCUGAGUG- 3'

**TABLE 6:** Information of forward, reverse, and sequencing primers of each knockout gene

Gene	Forward primer	Reverse primer	Sequencing primer
ADK	5′-TGAAATCCCATTCATAACAGCTACA-3'	5′-TGCCTCATGAATTCAACTGCA- 3'	5′-CATTCATAACAGCTACAGTTTCTCACAAAG-3'
AK1	5′-CCTGGTTTTCCTCCTCACCC-3'	5′-ACTCAGTCCAGGTCTCCACA-3'	5′-CTCACCCACACTCCATGAATCAG-3'
AK3	5′-TCAGCAGCCCGAGGTCT-3'	5′-GAGAGGCGGACGCAACA-3'	5′-TCCCGGGCGGCGGGAGGGAAATGCCGCGCC-3'
AK4	5′-GGCAATGGCTTCCAAACTCC-3'	5′-CTAGTTCTCGTTCCCTCGGC-3'	5′-TGCGCGCGGTCATCCTCGGGCCGCCCGGCT-3'
AK5	5′-GAGGCTGAGGCAGGAGAATC-3'	5′-AGCCTCTAGTGAATTGTTGGACA-3'	5′-GAGAATCACTTGAACCAAGGAGGTAGAAG-3'
NME1	5′-TTGGGTTTTTACCAGAGGGAG-3'	5′-CCGTAGCAATGCACCTCCTA-3'	5′-CTGACCTGACAGATTTAAACCAGCTTTAT-3'
NME4	5′-ATCCCTGTCCCTTCCAGAGT-3'	5′-ACCTTGCAGATAACACCAGCA-3'	5′-CTTCCAGAGTGCCCTGCATAG-3'
NME6	5′-TGCTTTGGGTCCTTTCTTACA-3'	5′-CTCCCTTGATCCCATGAGCC-3'	5′-AAGTCTCCAATGGATTCTTAGTCTCCTTAG-3'
NME7	5′-TCCACCCTAAGTCACCAGTGA-3'	5′-AGCCACTGCAAGACACTCAA-3'	5′-CCAGTGATAGAAATGCTTTCCTTTGATATA-3'
PKLR	5′-CCGACTCTGGACCCTAAGGA-3'	5′-GCGCCTCAAGGAGATGATCA-3'	5′-CTAAGGAGGGAGCCAGAGGAGATG-3'

### Metabolism of GS-441524 and ODV in single-gene knockout A549-hACE2 cells

After seeding 200,000 cells of WT and 200,000–300,000 knockout cells in 12-well plates overnight, these cells were treated with the prodrug for 2, 6, and 24 hours. At the end of treatment, cells were washed with 1x DPBS once, and 500 µL of 70% methanol (Quench Buffer) was added to each well. The 12-well plates were frozen at −80°C overnight. The cells were collected the next day by scraping down and collecting them in a 1.5 mL centrifuge tube. To normalize the metabolite formation with cell number, cells from an extra well were collected at each time point by trypsinization and quantified on a Nexcelom cell counter.

### Antiviral activity of nucleoside/tides against SARS-CoV-2 and CC_50_ in single-gene knockout A549-hACE2 cells

The antiviral effect of the tested compounds was evaluated in A549-hACE2 WT, A549-hACE2 ADK (KO), and A549-hACE2 AK2 (KO) cells. The virus (SARS-CoV-2 WT, expressing the gene for Nano-luciferase) was obtained from the UTMB. The infection was conducted in DMEM, supplemented with 1× Pen-Strep-Glutamine, and 2% FBS. The test compounds, DMSO (negative control) or nirmatrelvir (positive control), were spotted at two hundred nanoliters per well using an Echo acoustic dispenser (Labcyte, Sunnyvale, CA, USA) at the respective concentrations. The cells were seeded on the pre-spotted plates at 5,000 cells per well in 30 µL of assay medium. SARS-CoV-2-NLuc was added to the cells at an MOI of 0.35 in 10 µL, and the infected cells were incubated at 37°C, 5% CO_2_ for 48 hours. The final read (luminescence) was obtained using Nano-Glo Luciferase Assay System from Promega (N1150), and the signals were measured using an Envision microplate reader (Revvity). Dose-response curves were fitted based on a non-linear regression model. All calculations were performed using GraphPad Prism 8. Data represented the means of four independent biological experiments.

The cytotoxic effect of the compounds on the A549-hACE2 WT and A549-hACE2 AK2 *KO* cells was determined. The test compounds were spotted as described in the prior section, using an Echo acoustic dispenser (Labcyte, Sunnyvale, CA, USA). A549-hACE2 cells or A594-hACE2 AK2 *KO* were seeded into the pre-spotted assay plates at a density of 5,000 cells per well with the same media as for the infection. The assay plates were incubated for 2 days at 37°C and 5% CO_2_. At the end of incubation, CellTiter-Glo reagent (Promega, Madison, WI, USA) was added at an equal volume per well and incubated for 15 minutes. Then, the luminescence signal was read using an Envision. Puromycin was used as a positive control, and DMSO was used as a negative control.

### Statistical analysis methods

Statistical analysis was performed using GraphPad Prism version 10.6. Statistical significance was determined using *P*-values calculated by multiple unpaired t tests. Data are presented as mean ± standard deviation. A *P* value of < 0.05 was considered statistically significant. Statistical significance for multiple comparisons was corrected with the Holm-Šídák method using an alpha factor of 0.05.
